# Book Reviews

**Published:** 1904-12

**Authors:** 


The Principles and Practice of Gynecology. For Students and Practitioners. By E. C. Dudley, A.M., M.D., President of the American Gynecological Society; Professor of Gynecology, Northwestern University Medical School, Chicago. Fourth Edition. Octavo, pp. 771. With 419 illustrations in colors and monochrome, of which 18 are full-page plates. Philadelphia and New York: Lea Brothers & Company. 1904. (Price: cloth, $5.00; leather, $6.00; half morocco, $6.50 net.)

When the first edition of this admirable treatise was sent out it commanded attention immediately, and very soon won the favor of teachers, practitioners and students; so much so, indeed, that without disparagement to others, it may be termed with great propriety the leading American textbook on gynecology. But if this is to be said of the book as the sum of its first three editions, what is there left for remark regarding this—the fourth?

Briefly summarised, it has undergone a thorough revision which has served to incorporate all that is new of value in the text. This has rendered it necessary to rewrite many chapters, to rearrange others, and to reduce still others, until now the volume is as nearly an exponent of the gynecology of the present day, as it is possible for language to express in print. We are fully justified in accentuating this point, because the author is a master of correct English, including compactness of style and forcefulness of expression.

We need not take up this book in detail for it is already familiar to every gynecologist, as regards its general plan and its methods of dealing with the various topics, but we were impressed when it first appeared with the practical, scientific and systematic way in which the author dealt with the subject of gynecologic diagnosis. And now, with the additions and revisions that appear in this edition, we are more than ever of the opinion that it is the best delineation of this topic that has yet been published in a textbook. It is so important to acquire the habit of diagnostic precision as well as to attain diagnostic acumen, that it will repay the student and junior physician to study Dudley's methods most diligently, if they would become proficient in gynecological work.

There is another one of the many excellent features of this work, to which we would invite special attention—namely, the wealth of illustration that adorns its pages. The author has made technical study of the art of illustration, being possessed himself of an artistic eye, hence has been able to develop the greatest perfection in the pictorial presentation of gynecological art. More than three hundred new pictures have been added to this edition, all from drawings made specially for this purpose; indeed, we may congratulate the author on eliminating all borrowed illustrations, so that now every picture in the book is distinctly original. This gives the treatise a personality that does not belong to any other similar one and, besides, increases its value as a teaching medium.

The most important feature of the illustrations, and this is one in which the author takes special pride, is that all minor and major operations and procedures are shown in their several steps, beginning oftentimes with the initial cut and continuing through the several stages, until the last stitch is tied. For example, there are thirty-two drawings in explanation of perineal lacerations and the methods of their repair, while the various steps of hysteromyomectomy are described most graphically by twelve drawings.

The author of this work is regarded,—very properly so, too,—as one of the leading gynecologists of the day, and being congenitally possessed, withal, of an inquiring mind, it is not strange that he should have constructed a book without a rival as a guide in the lecture room and in the clinic.

---

Transactions of the Medical Society of the State of New York, Ninety-Eighth Annual Session, held at Albany, January 26, 27, 28, 1904. Frederic C. Curtis, M.D., Secretary. Published by the Society. 1904.

The annual meeting which this book records ever will be distinguished by the fact that it placed its unanimous approval upon the report of the committee of conference which, for two years previously, had been engaged in an effort to unite the Medical Society of the State of New York and the New York State Medical Association. It is a significant fact that notwithstanding differences of opinion not only as to details, but also in the minds of some as to expediency, these were all harmonised and the report was unanimously confirmed, not only by the state medical society, but by all the county medical societies in affiliation with it. That there is failure to bring about union is regrettable; but it is a subject of congratulation that such failure was not due to any action of the Medical Society of the State of New York nor to that of any of its affiliating county societies, nor, again, to any member of either, acting in his individual capacity. All of the responsibility for this failure of union therefore must rest with the New York State Medical Association and its affiliated county organisations.

This volume is replete with excellent papers, one of which is by Dr. Arthur Twining Hadley, president of Yale University, entitled, The conflicting claims of general education and professional education. The president of the society, Dr. Algernon T. Bristow, chose as the subject of his anniversary address, The present status of the medical expert. A symposium on diabetes brought out as participants Richard M. Pearce, David L. Edsall, William H. Thompson, Frederic C. Shattuck and Heinrich Stern. A symposium on nephritis was participated in by Francis Delafield, Joshua M. Van Cott and Beverly Robinson. A symposium on abdominal pain was presented by John H. Musser and Frederick Holme Wiggin. A symposium on typhoid fever brought together as participants Henry A. Fairbairn, Henry L. Elsner, Egbert Le Fevre, Luzerne Coville and Cyrus W. Field. Finally, a Röntgen ray symposium included as participants Arthur Dean Bevan, Charles Lester Leonard, William B. Coley and Hon. W. W. Goodrich.

The official stenographer, Dr. Ogden C. Ludlow, of New York, died before his minutes were fully transcribed, hence the discussions are considerably curtailed.

In the minutes of the proceedings are to be found the usual reports of committees and officers, among which is that of the State Board of Medical Examiners. It is difficult to understand why this board, the most important creation of the society, should be compelled each year to present its report in abstract. The excuse is always that there is lack of space, and also there is manifested an indisposition to incur the necessary expense. Neither of these reasons would seem to comport with the dignity of this great society which has done so much to advance medical educational in this country, and which should not hesitate to spread the results annually before the country.

---

The Surgery of the Heart and Lungs. By Benjamin Merrill Ricketts, Ph.B., M.D., Cincinnati. Octavo, pp. 526. Illustrated. New York: The Grafton Press. 1904.

The author of this work has presented subjects of growing interest to all surgeons. It is scarcely ten years since it was deemed possible to attack the heart for the relief of penetrating wounds; pulmonary surgery, however, has had a longer career, and yet only lately has it become of recognised value. We have been impressed with the painstaking character of the work performed by the author in preparing this book. It has involved strenuous labor and will live as a pioneer treatise of inestimable value on the subjects of which it treats.

The anatomy of the heart, as given by Ricketts, is highly interesting and, withal, is one of the most complete studies of the structure of the organ we have seen. The researches in comparative anatomy deserve special mention, as also does the section on the mechanics of the heart-beat. This entire chapter could be studied with profit by every student of anatomy.

Gunshot, lacerated, and incised wounds of the heart, though not common, are of such comparative frequent occurrence as to make interesting studies relating to their surgical repair. A number of cases have been reported in which suture of the heart has been followed by recovery, reference to these being made in this book. In 1871, Callender removed a needle from the heart, which is the first recorded instance of an attempt at surgical relief for heart injury. In 1877, Roswell Park, in attempting to relieve pericardial effusion by aspiration, by accident thrust the needle into a myocardial abscess, and in this manner removed several ounces of pus, thereby affording the patient temporary relief,—probably the first instance in which surgical aid was involved for disease of the heart structures.

The surgery of the lungs, though antedating that of the heart is, nevertheless, of absorbing interest, and the literature contains many instances of surgical cure of injuries and diseases of the lower respiratory tract. Ricketts has collected much of historical interest on the surgery of the lungs. The removal of foreign bodies from the lung or bronchial tubes is frequently done, and lung surgery for disease is more common than for traumatism.

The illustrations are an interesting feature of this work, and very properly begin with a frontispiece of the heart and lungs of a dog in situ. The anterior view of the heart from Deaver's surgical anatomy is a beautifully executed plate, and makes an appropriate beginning to the section on the heart. It is a work that will be found of great use to the surgical profession, and many physicians who do not practise surgery will be interested in its valuable contents.

We could wish the author had found occasion to use diphthongs with less freedom, thus making the text conform to more modern usages; for example, "sæptum" is an archaism that offends the eye in the extreme.

---

Transactions of the American Association of Obstetricians and Gynecologists. William Warren Potter, M.D., Secretary. Volume XVI. For the year 1903. New York: Rooney & Otten Company, Printers.

Whoever will undertake the examination of this book cannot fail to be impressed with the conscientious nature of the work done by this association at the annual meeting of which record is herein made. Many of the papers are of more than ordinary importance, while the discussions develop a strength that accentuates their value to unusual degree.

Chicago is an excellent city to play the host to such a body; it is full of able physicians, who take interest in such gatherings, and who manifest their interest by attendance on the sessions, by participating in the debates, and by extending various hospitalities that serve to stimulate the members to their best efforts.

The paper of Dr. John B. Murphy on Tuberculosis of the female genitalia and peritorieum, which occupies eighty pages of the book, is one of unusual interest, and must have cost the author years of research and observation. It is the most considerable contribution to the literature of the subject that has yet been made, and will be quoted authoritatively for years.

Many other papers are of great value, but they should be read rather than described. It is doubtful if the association has ever put forth a more comprehensive or more practical volume than this, which is saying a good deal of a book that has so many noted companions.

---

The Practice of Obstetrics. Designed for the Use of Students and Practitioners of Medicine. By J. Clifton Edgar, Professor of Obstetrics and Clinical Midwifery in the Cornell University Medical College, New York. Royal octavo, pp. 1153. With 1264 illustrations, including 5 colored plates and 38 figures printed in colors. Second edition, revised. Philadelphia: P. Blakiston's Son & Company. 1904. (Price, $6.00 net.)

We can quite comprehend how such an excellent treatise as this should become promptly, not to say suddenly, exhausted in its first edition. This is the first instance, we believe, in the history of the Journal, in which two editions of the same work on obstetrics have been reviewed within a single year. But we do not intend to review this second edition; coming so soon after the first, it is quite unnecessary to do so. However, we are glad to have an opportunity to invite particular attention to a few important features of the book that were not touched upon in the first instance.

One of the misunderstood details of the management of the third stage of labor by probably about one third of the candidates for the New York state license is Credé's method of delivering the placenta. In a normal labor,—normal as to first and second stages,—the conditions may change from calm to storm if the obstetrical attendant is not perfectly familiar with placental expression. Edgar describes Credé's plan in exceptionally simple phraseology making it so plain that "none but a fool may err therein." He says: "To practise this the fundus is grasped with one hand, fingers behind and thumb in front, and a contraction awaited. At the height of the pain the uterus is firmly compressed and forced downward and backward into the pelvis. If the first attempt fail, another may be made in the same manner at the next contraction. It may be necessary to repeat this procedure during several contractions." This, and this only, is Credé's method of placental expression,—a fact that should be drilled into every medical student, until he thoroughly understands it and can practise it with skill.

The extraction of the after-coming head is another piece of technic that should be made plain to the student of obstetrics. He can get such instruction on this subject in this book, as will enable him to deal with this complication in a clear and understanding way.

Again, we desire to call attention to the illustrations, 1,259 in number, most of which are original and all of which are a credit to this modern obstetric treatise.

---

Transactions of the Southern Surgical and Gynecological Association. Sixteenth annual meeting held at Atlanta, Ga., December 15, 16 and 17, 1903. William D. Haggard, M.D., Secretary. Philadelphia: William J. Dornan, Printer.

If the transactions of special societies were read more universally by general practitioners of medicine the aggregate stock of knowledge would be very much increased. In many such societies and particularly in the Southern Surgical and Gynecological Association questions are often determined that have been regarded doubtful until discussed in this body. The present volume is a splendid tribute to the men who participated in the proceedings of the last annual meeting which was held at Atlanta.

The interesting case of Bilateral diffuse virginal hypertrophy of the breasts, by George Ben Johnston, merits special notice because first, of its rarity, and second, on account of the careful preparation of the paper which discusses the pathology of the anatomical anomaly and gives bibliographical references that serve to make a completeness to the paper which is most satisfactory. The illustrations also are to be commended.

The presidential address of Dr. Bovée, which dealt with Problems in urethral surgery, is an excellent presentation of these difficult questions. The general makeup of the book, which is carefully edited by the secretary, deserves special commendation.

---

A Practical Treatise of Diseases of the Skin. For the Use of Students and Practitioners. By James Nevins Hyde, A.M., M.D., Professor of Skin, Genitourinary and Venereal Diseases, Rush Medical College, Chicago, and Frank Hugh Montgomery, Associate Professor of Skin, Genitourinary and Venereal Diseases, Rush Medical College. Seventh and revised edition. Octavo, pp. 938. Illustrated with 107 engravings and 34 plates in colors and monochromes. Philadelphia and New York: Lea Brothers & Company. 1904. (Price: cloth, $4.50 net; leather, $5.50 net.)

For many years this treatise has been recognised as authoritative among teachers and specialists in dermatology. Since it was first issued,—namely, in 1883, then, however, solely under the name of the senior author,—it has stood for the best and among the best, in dermatological literature. Since 1893 it has appeared in new editions on an average of once in three years or a trifle less, always offering the latest and more approved methods of treatment, besides teaching the most assured methods of diagnosis.

The revision that became necessary when this seventh edition was projected resulted in a thorough overhauling of the entire work, and the revision proceeded page by page until completed; hence, for all practical purposes this is a new work, with this substantial difference, that it is prepared by men of experience who are familiar with all the details of authorship. This makes for substantial gain over the average new book without a previous record.

The illustrations are of a high class and depict with accuracy many conditions that are often difficult of diagnosis. Color has been used to good advantage in numerous instances. It is a book that reflects the present status of dermatological science, and will maintain the high place it has already attained, as an exponent of the practice of a sometimes difficult but always important branch of medicine.

A System of Practical Surgery. By Drs. E. von Bergmann, Berlin, P von Bruns, Tübingen, and J. von Mikulicz, Breslau. Volume IV. Surgery of the Alimentary Tract. Translated and edited by William T. Bull, M.D., Professor of Surgery, College of Physicians and Surgeons, Columbia University, New York. Pages 757. Illustrated. To be complete in five imperial volumes. Philadelphia and New York: Lea Brothers & Company. 1904. (Price, per volume: cloth, $6.00; leather, $7.00; half morocco, $8.50.)

The American medical profession is to be congratulated in being able to possess a work that concededly stands as the foremost exponent of present day surgery on the continent of Europe. Dr. William T. Bull and his assistants, Foote, Flint and Martin, have translated and edited von Bergmann's treatise so promptly that scarcely more than a year has elapsed since it was first announced. Moreover, the translation is so perfectly made that none of the sense of the original is lost or impaired.

The present volume, number four, deals with malformations, injuries and diseases of the esophagus; injuries and diseases of the abdominal wall; of the peritoneum, including laparatomy; malformations, injuries and diseases of the stomach and intestine, including hernia; injuries and diseases of the liver and biliary passages; of the spleen; and of the pancreas.

From the foregoing it will be observed that this volume includes the surgery of the entire alimentary tract, of the abdominal viscera, and of hernia. It is illustrated with 345 engravings and is a volume that no surgeon can afford to do without.

---

International Clinics. A Quarterly of Illustrated Clinical Lectures and especially prepared articles on Treatment, Medicine, Surgery, Neurology, Pediatrics, Obstetrics, Gynecology, Orthopedics, Pathology, Dermatology, Ophthalmology, Otology, Rhinology, Laryngology, Hygiene and other topics of interest to students and practitioners. By leading members of the medical profession throughout the world. Edited by A. O. J. Kelly, A.M., M.D., Philadelphia. Volume III. Fourteenth series. Philadelphia: J. B. Lippincott Company. 1904. (Cloth, $2.00.)

The foreign contributors to this number include Allchin, Duncan, Lockyer, Stewart and Williams, of London; Ballantyne, of Edinburgh; Boas, of Berlin; Carriere, of Lille (France); Chauffard, Fournier, Gouraud, Guisez, Lermoyez and Milian, of Paris. The American contributors are Bishop, Gottheil, Hart, Katzenbach, and Manley, of New York; Brown, of Saranac Lake; Cumston and Davenport, of Boston; Langdon and Palmer, of Cincinnati; Neilson and Spiller, of Philadelphia; and Ohmann-Dumesnil, of Saint Louis.

The subjects dealt with are, syphilis, twelve articles; treatment, three articles; medicine, four articles; surgery, four articles; gynecology, three articles; and neurology, one article.

Manley discusses the important subject of strangulated umbilical hernia in the female, which is well illustrated. Other articles of absorbing interest are also presented, some of which are illustrated, and the entire volume is one of the most instructive of the series.

---

Progressive Medicine. Volume III, September, 1904. A Quarterly Digest of Advances, Discoveries and Improvements in the Medical and Surgical Sciences. Edited by Hobart Amory Hare, M.D., Professor of Therapeutics and Materia Medica in the Jefferson Medical College of Philadelphia. Octavo, 284 pages, 19 illustrations. Philadelphia and New York: Lea Brothers & Company, Publishers. (Per annum, in four cloth-bound volumes, $9.00; in paper binding, $6.00, carriage paid to any address.)

The contents of this volume embraces four general topics, the literature of which has been condensed and presented in abstract. It concerns chiefly the journal articles for 1903, but in a few instances it includes those published in the early part of 1904, and in still fewer instances some of those published in 1902.

The section of diseases of the thorax and its viscera, including the heart, lungs, and bloodvessels, is prepared by William Ewert, of London; the section of dermatology and syphilis, by William S. Gottheil; the section on diseases of the nervous system is compiled by William G. Spiller; and the section on obstetrics is abstracted by Richard C. Norris. The usual index concludes an interesting digest of these several topics.

---

Practical Materia Medica for Nurses. By Emily A. M. Stoney, Superintendent of the Training School for Nurses in the Carney Hospital, South Boston, Mass. Duodecimo, 300 pages. Second edition, thoroughly revised. Philadelphia, New York, London: W. B. Saunders & Company. 1904. (Cloth, $1.50 net.)

Something over five years ago we noticed in these columns the first edition of this useful book. We are glad to learn that it has been sufficiently appreciated by nurses to exhaust the previous publication, and to justify the printing of a new edition. It contains such information as every nurse needs, regarding the source, action, and use of drugs, together with the symptoms and treatment of poisoning; besides, there is added a glossary of terms used in materia medica. All things considered, it is one of the most useful books that a nurse can add to her library. The revision has included all the newer drugs of value, and many other notable features.

---

An Introduction to Vertebrate Embryology, Based on the Study of the Frog and the Chick. By Albert Moore Reese, Ph.D., Associate Professor of Histology in Syracuse University. Duodecimo, pp. 291. Illustrated. New York and London: G. P. Putnam's Sons. 1904.

This author modestly disclaims originality for his book, regarding it merely a compilation. It is, nevertheless, one of the most useful aids to the study of embryology that has appeared up to the present time. It presents an outline of the essentials concerning the development of the chick and the frog,—the two animals that are commonly studied by medical students. Indeed, the book has been prepared with a view to the special requirements of students. The subject is dealt with in a fascinating manner and would afford interesting reading for any person of intelligence. It is well illustrated by a clear set of diagrams and engravings on wood and is a most admirable presentation of elementary studies in embryology.

---

The Medical Directory of New York, New Jersey and Connecticut. Published by the New York State Medical Association. Volume VI. 1904-1905.

If a medical directory could speak out and tell how it was compiled, perhaps it would lead to a greater degree of accuracy. The one before us is as accurate as any, and we presume that an adequate reason for whatever imperfections it contains is to be found in the carelessness or indifference of physicians themselves.

The total number of names of physicians contained in the lists published in this volume is 15,204, of which 11,746 reside in New York state; 2,176 in New Jersey; and 1,282 in Connecticut. Of the 11,746 physicians in New York state, 4,215 are credited to Manhattan and Bronx, 1,506 to Brooklyn, 121 to Queens, 65 to Richmond, a total for Greater New York of 5,907, which makes the number of physicians residing in the rest of the state, 5,839.

---

The Gazette Pocket Speller and Definer. English and Medical. Second edition. New York: The Gazette Publishing Company. 1904. (Price, 50 cents.)

It is often convenient to have at hand a word book of small size for quick reference. This is just such an one, and it even can be carried in the pocket,—the vest pocket if need be. It occupies a field by itself and is all that it aims to be—namely, a compact speller and definer of English and medical words, for ready reference.

---

A Textbook of Mechano-Therapy (Massage and Medical Gymnastics) for Medical Students, Trained Nurses and Medical Gymnasts. By Axel V. Grafstrom, B.Sc., M.D., Attending Physician to the Gustavus Adolphus Orphanage, Jamestown, N. Y. Second edition, revised and enlarged. Duodecimo of 200 pages, illustrated. Philadelphia, New York, London: W. B. Saunders & Company. 1904. ($1.25 net.)

The author of this manual is an expert in the application of movements to the treatment of disease, and in medical gymnastics. His announced purpose when the book was issued first, now some five years ago, was to make it a rational textbook for the student, nurse, and medical gymnast. That he has succeeded in accomplishing his purpose, at least to a very large extent, is testified to by the demand for a second edition. There has been considerable revision of this book, as well as some additions to it, which make it as complete a work of reference for a physician as could be prepared for the purposes indicated.

---

The Optical Dictionary. Edited by Charles Hyatt-Woolf, F.R.P.S., Editor of "The Optician and Photographic Trades Review." Philadelphia: P. Blakiston's Son & Company. 1904. (Price, $1.00.)

As a matter of convenience to students and junior physicians it is well to have a separate dictionary of optical terms,—for example, one like this,—that can remain within easy reach on the desk or library table. Ophthalmological terms are more or less complex and this glossary will assist in fixing them upon the mind. The mechanical work of its preparation is exceedingly well done, though its imperfections are many.

---

Visiting Lists, 1905.

Notices of the following Visiting Lists are given in the order in which they were received. Each has its special merits of which our readers must judge either from experience or description.

some being adapted to the needs of one and some to those of another. All, however, are of superior excellence, the main points of difference being such as we shall endeavor to present in the description which follows:

The Physicians' Visiting List (Lindsay and Blakiston's) for 1905. Philadelphia: P. Blakiston's Son & Company, 1012 Walnut Street. Sold by all booksellers and druggists.

The Lindsay and Blakiston Visiting List, now in the fifty-fourth year of its publication, contains the essentials which most physicians need and is a time-saving device that has met approval for over half a century. It is bound in handsome half morocco, is equipped with a rubber tipped pencil, is of convenient shape and size, is made of strong thin gilt edged paper and contains useful memoranda for office or bedside reference. Among the latter the subject of incompatibility is discussed, the immediate treatment of poisoning is tabulated, the metric system and equivalents are given, besides which there is a dose-table of drugs, an obstetric table and numerous other clinical data are given. In our advertising pages will be found a list of sizes and prices.

---

The Medical News Visiting List for 1905. Lea Brothers & Company, Philadelphia, 706 Samson Street; New York, 111 Fifth Avenue.

The Medical News Visiting List has become a standard record for the profession. It is published in four different styles with the object of adjusting itself to the requirements of the largest number of physicians.

It contains much clinical material and data of value for immediate reference, among which we may mention tables of weights and measures and comparative scales; instructions for examining the urine; table of eruptive fevers; incompatibles, poisons and antidotes; directions for effecting artificial respiration; extensive table of doses; an alphabetical table of diseases and their remedies, and directions for ligation of arteries.

It is bound in strong leather cover, wine tinted in color, contains a pocket, a pencil with eraser and 192 pages of well-ruled fine gilt edged paper.

The weekly, monthly and 30-patient perpetual contain 32 pages of data and 100 pages of classified blanks. The 60-patient perpetual consists of 256 pages of blanks alone. Each is made up in one wallet-shaped book, bound in flexible leather, with flap and pocket, pencil and rubber, and calendar for two years, $1.25. Thumb-letter index, 25 cents extra. By mail, postpaid, to any address.

---

The Medical Record Visiting List or Physicians' Diary for 1905. New revised edition. New York: William Wood & Company.

The Medical Record Visiting List has always been a favorite with a large number of physicians. It ranges in price from $1.25

to $4.00, according to size, style and quality of binding. It is a compact book, easily adapted to the morning coat pocket and contains material for ready reference adapted to emergencies as well as to the ordinary daily practice of a busy physician. It has an obstetric calendar, deals with weights and measures, gives tables of doses, the treatment of poisoning, hints on writing wills, addresses of nurses, besides many other practical hints. It is printed on excellent paper with gilt edges and is bound in fine black leather which contains a pocket and pencil.

